# Psychiatric symptoms at age 8 as predictors of specialized health service use for psychiatric disorders in late adolescence and early adulthood: findings from the Finnish Nationwide 1981 Birth Cohort Study

**DOI:** 10.3389/fpsyt.2025.1600022

**Published:** 2025-07-01

**Authors:** Andre Sourander, Katri Kaajalaakso, Miika Vuori, Lauri Sillanmäki, Terhi Luntamo

**Affiliations:** ^1^ Department of Child Psychiatry, University of Turku and Turku University Hospital, Turku, Finland; ^2^ Research Centre for Child Psychiatry, University of Turku, Turku, Finland; ^3^ INVEST Research Flagship Center, University of Turku, Turku, Finland; ^4^ Research Unit, The Social Insurance Institution of Finland (Kela), Helsinki, Finland

**Keywords:** epidemiology, follow-up, childhood, predictors, psychiatric disorder

## Abstract

**Background:**

A majority of adult psychiatric patients have suffered of psychiatric symptoms already during childhood or adolescence. Previous studies on continuity of mental health problems have included only children with diagnoses and thus missed symptomatic children without mental health service contact, or as in longitudinal cohort studies, have suffered from considerable losses of subjects during follow-up. The aim of the present study was to examine the association between multi-informant ratings of childhood psychiatric symptoms and cumulative incidences of diagnosed mental disorders in early adulthood in a large population-based cohort.

**Methods:**

The original sample consisted of 6,017 Finnish children born in 1981. Parents and teachers completed the Rutter questionnaire, while the children completed the Children’s Depression Inventory (CDI) at the age of 8. The follow-up information was obtained from the Finnish Care Register for Health Care. Analyses were done with the Cox regression model.

**Results:**

A total of 2,717 males (12.4%) and 2,683 females (12.8%) were diagnosed with a mental disorder in early adulthood. Parent-/teacher-reported high levels (above the 90th percentile) of conduct problems (males, Hazard Ratio (HR)=2.5, 95% CI=1.6-3.7; females, HR=1.5, 95% CI=1.01-2.1) and anxiety (males, HR=1.5, 95% CI=1.1-2.1, females, HR=1.7, 95% CI=1.2-2.4), and child self-reported depressive problems (males, HR=1.7, 95% CI=1.2-2.3; females, HR=1.6, 95% CI=1.1-2.2) were key predictors of any mental disorder in early adulthood. Conduct problems predicted psychotic disorders and substance-related disorders among males. Anxiety problems predicted psychotic disorders, anxiety, and depression. Child self-reported depressive problems predicted male depression. Attention-deficit hyperactivity disorder (ADHD) symptoms and low school performance did not predict any outcome in multivariate analyses. A non-nuclear family living situation at age 8 predicted most outcomes.

**Conclusions:**

Adult-reported conduct and emotional problems in children, but not ADHD symptoms, independently predicted mental health service use and any psychiatric diagnosis in late adolescence and early adulthood, emphasizing the need for early identification of childhood mental health problems. Similarly, child self-reports of depressive problems already at age 8 predicted adult outcomes. Our findings emphasize the importance of multi-informant assessment and early targeted interventions for conduct and emotional problems in early school years.

## 1 Introduction

Mounting evidence shows that psychopathology in childhood increases the risk for mental disorders in adulthood (1–4). In a birth cohort study published in 2003, Kim-Cohen et al. (5) reported that, of the adults who received intensive mental health care, 77.9% had received a psychiatric diagnosis before 18 years of age, and 60.3% before 15 years of age. According to a meta-analysis by Solmi et al. (6), the peak age for the onset of mental disorders is 14.5 years. These findings show that a majority of adult psychiatric patients have suffered of psychiatric symptoms in their childhood and adolescence, indicating that continuity among these disorders is considerable.

Continuity among psychiatric disorders does not only refer to the persistence of symptoms until adulthood. Research has shown that a childhood disorder can precede either the same disorder in its adult form (homotypic continuity) or a different disorder (heterotypic continuity). For example, childhood conduct disorder has been shown to strongly predict adult conduct problems and antisocial personality (1–3, 5, 7), and it is known that childhood depression often continues into adulthood (4, 5, 8). These trajectories represent homotypic continuity. On the other hand, several studies have shown that conduct disorder also predicts depression and anxiety in adulthood (1, 3, 5, 7, 9), and childhood depression and anxiety cross-predict each other (3–5, 7, 8) as well as bipolar disorder (10), indicating heterotypic continuity among these disorders. In the ALSPAC study, it was found that individuals who displayed early-onset persistent conduct problems throughout childhood were at greater risk “for almost all forms of later problems” (11).

During recent decades, the prevalence of psychiatric service use and childhood predictors of different disorders have been widely studied, providing empirical knowledge about developmental trajectories, child mental health associations with later adversities and unmet needs of early interventions. However, interpreting the results of these studies can involve some limitations. First, if the information about childhood psychopathology is obtained from registers only, individuals who would have needed services in childhood, but did not receive them, may not be accurately represented. On the other hand, longitudinal cohort studies provide information about the population-based rates of problems, but during the follow-ups, sample losses are often high and cause significant bias. Third, most population-based cohort studies evaluate childhood symptoms based on one informing perspective, usually that of the parents, while birth cohorts that include self-reports from parents, teachers, and children when the children are in early school years are rare.

The present birth cohort study draws from a cross-sectional epidemiological study based on parent, teacher and child reports from when the child was 8 years old and the monitoring of the individual’s specialized service use during late adolescence and early adulthood. The baseline data with a 97% participation rate is representative of Finland as a whole, and its linkage with a well-validated nationwide Finnish register data allows the unique possibility to examine childhood predictors of psychiatric service use. There are several important ongoing Nordic register studies, and some of them also include linkage with questionnaire (12–15). When compared to previous literature, the strengths of the present study were: 1) the sample is representative and nationwide; 2) the baseline sample has a very low attrition rate; 3) the nationwide questionnaire data is based on ratings by parents, teachers and children at a very early age of the child, i.e. when they were 8-year-olds; 4) the multi-informant questionnaire data has been linked with available national register data in adolescence and early adulthood, until age 29.

The aim of this study is to examine how childhood psychiatric problems predict specialized service use and psychiatric diagnoses. Our hypothesis, based on previous research, is that childhood conduct problems are strongly associated with service use and a wide range of psychiatric problems. We expect that internalizing problems show homotypic continuation.

## 2 Materials and methods

### 2.1 Participants

The study is a part of the multicenter “Finnish Nationwide 1981 Birth Cohort Study” (16). The population of the study consisted of all 60,007 Finnish children born in 1981 who were alive and living in Finland in 1989. A random sample of 6,017 children were invited to take part in the study in 1989, at the age of 8–9, representing approximately 10% of the population. Participants were recruited from all five university hospital areas in Finland (Helsinki, Turku, Tampere, Kuopio, and Oulu). The sample reflects good generalizability regarding the population (16).

Study procedures were implemented through the schools in November 1989, when the subjects attended the second grade in elementary school. Information was collected from three different sources: parents, teachers, and children. The study material was delivered to the teachers, who sent the parent questionnaires and information sheets via the children to their parents. The parents returned the questionnaire to the teacher in a sealed envelope. The participating children completed the questionnaire in a classroom setting. Thereafter, the teacher sent both child and parent questionnaires as well as the teacher questionnaires to the researchers.

**Figure 1:**
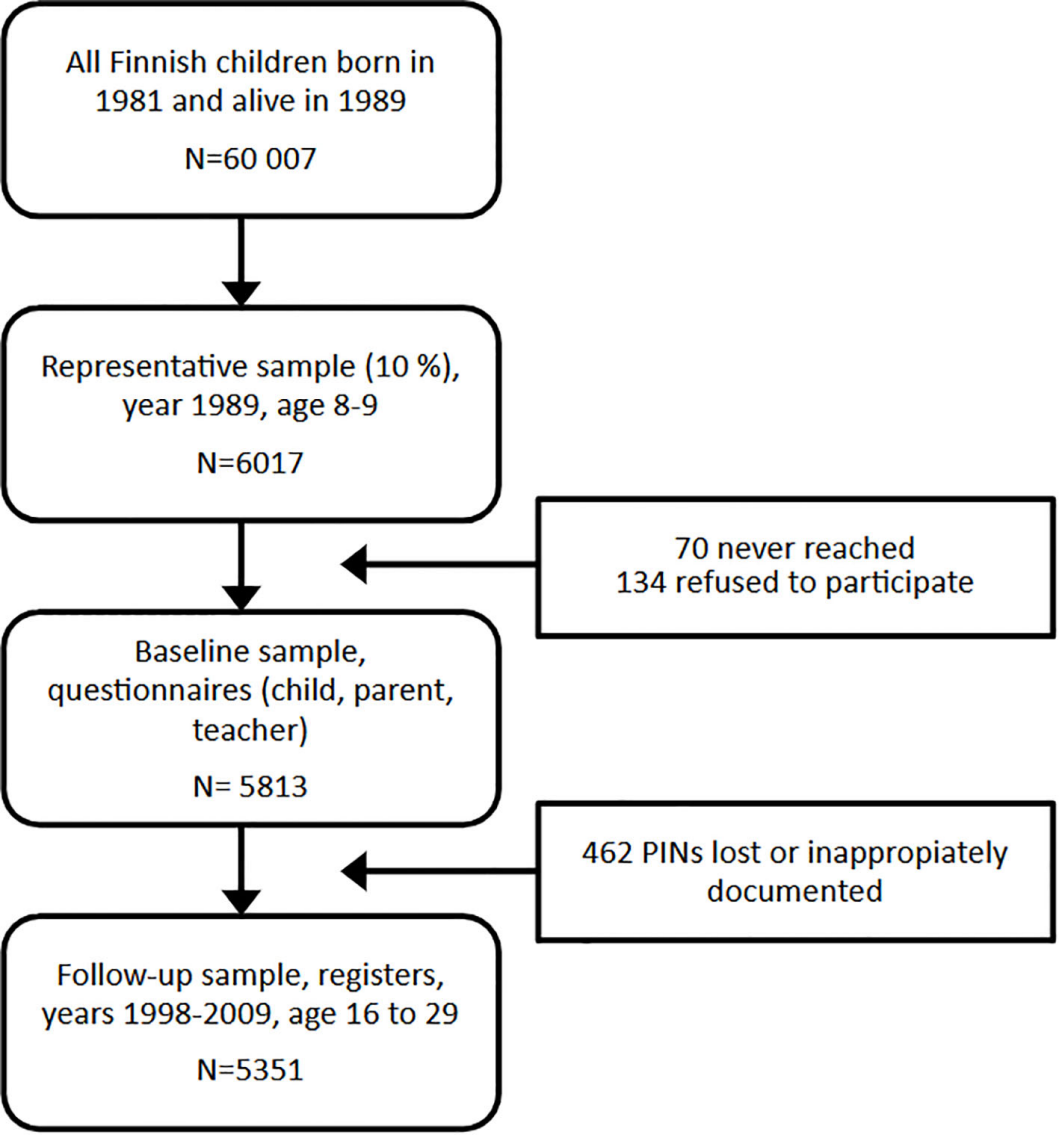
Flow chart of the study participants. PIN, personal identification number.

Of the selected 6,017 children, 5,813 (96.7%) took part in the study in 1989. Individuals who are born Finnish citizens are assigned a unique Personal identification Number (PIN) at birth, and this number stays the same throughout their life, regardless of changes of name or place of residence. In this study sample from 1998 to 2009, the personal PINs of 5351 participants were linked with the Population register and the Care Register for Health Care. However, when the linkage was done 10 years after baseline collection, 7.7% (462 out of 6014) of PIN codes had not been documented at all or had been documented inappropriately. Therefore, these PIN codes were missing at random. The sample which was linked to registers was 5351 subjects (89.0% of the original 6014) (**Figure 1**).

### 2.2 Ethics approval and consent to participate

The Joint Commission on Ethics of Turku University and Turku University Central Hospital, as well as the school authorities approved the research plan. The combined information from the questionnaires and the registry data was analyzed in such a way that no subject could be identified. Participation in the study was voluntary. The information sheet accompanied the questionnaire that was sent to the parents. According to the national regulations at that time, a completed parental questionnaire was considered to be informed consent for the child’s participation in the study.

### 2.3 Predictors at the age of eight

The study utilized a multi-informant approach (17). The Rutter’s parent questionnaire (RA2) (18) and teacher questionnaire (RB2) (19) were used to collect information from the parents and teachers. These questionnaires are commonly used in child psychiatric research (20). In previous Finnish studies, both scales have been validated (21), and the correlation between the parent and teacher scales has been shown to be moderate (22). The parent and teacher questionnaires include 31 and 26 items, respectively, and the answers are scored from 0 to 2 points. In addition to the total score scale, there are three subscales: the conduct scale, which screens for antisocial behavior; the ADHD (originally named hyperactivity) scale, which screens for restlessness and attention deficit symptoms; and the emotional scale (mostly measuring anxiety e.g. shyness, withdrawal, and other anxiety symptoms).

To measure child self-reported internalizing symptoms, the children completed the Children’s Depression Inventory (CDI) (23), which charts depressive symptoms between the ages of 7 and 17. The original English version of the CDI has shown good validity for assessing depressive symptoms among children (24) but insufficient discrimination from anxiety disorders (25). The CDI includes 27 items, which are scored from 0 to 2 points. In this study, the question concerning suicidal ideation was left out, based on the decision of the core research committee. It was considered unethical to include this question in the questionnaire, considering the young age of the participants. Therefore, the version used in this study had 26 items and a maximum score of 52 points.

The scores from the parent and teacher questionnaires were combined to generate pooled scores for the conduct, ADHD, and emotional subscales, whereas the total score for depression was based on the child self-report assessment alone. Because the ranges of potential scores for conduct, hyperactivity, and emotions were different for parents and teachers, the scales were standardized so that each informant would be given equal weight. The subscales used in this study were standardized separately within male and female samples. First, the subscales were standardized by subtracting the mean value of the scale from the observed value of the subject and dividing that by the standard deviation of the scale across raters. The pooled total subscale was created by combining the parent subscale scores with the corresponding teacher rating scores (26). If only the parent or the teacher information was available, but not both, the pooled scale was not generated for that subject. To generate easily interpretable measures of psychopathology, the results of the four scales were categorized as below the 50th percentile (absence of mental health problems), between the 50th and 90th percentile (low or moderate level of problems) and above the 90th percentile (high level of problems). Of note, the group below 50th percentile had 0 or 1 points in respective scales. As previous studies have found that even low or moderate levels of conduct problems and ADHD symptoms in childhood are associated with subsequent adverse outcomes, both low/moderate and high level of problems in childhood were analyzed in relation to the outcomes (27, 28). The cut-off points were sex-specific and based on the distribution of scores in the present sample.

Parents also answered questions about parental education level (12 years or more of education or a lower education level) and family structure (two biological parents or another constellation) as a part of the parent questionnaire. School performance was derived from the teacher report: answers depicting average and better than average performance were pooled and compared to that of poor performance.

### 2.4 Specialized service use for psychiatric disorders by the age of 29

The PIN codes of the participants were linked to data obtained from the Care Register for Health Care (CRHC) covering the period between 1 January 1998 and 31 December 2009, when the subjects were from 16 to 28 years old. Since 1998 the CRHC has included information from all visits in public outpatient clinics in Finland, not only inpatient data. For this reason, the follow-up started in 1998. The CRHC is maintained by the Institute of Health and Welfare, and it has been described in detail in previous publications (29, 30). In the CRHC, all visits are registered and diagnoses are made according to the International Classification of Diseases, the tenth revision (ICD-10). In this study, we only looked at main diagnoses. We included all subjects with outpatient visits or inpatient admissions in the register in the study, i.e. service use in both psychiatric and general hospitals was included. We focused on the following diagnostic groups: any psychiatric diagnosis (ICD-10 codes F00-F99); schizophrenia and other psychotic disorders (F20-F29); bipolar disorder (F30-F31); depression disorders (F32-F39); anxiety, stress-related, adjustment and somatoform disorders (F40-F48; abbreviated anxiety); eating disorders (F50); and substance use disorders (F10-F19).

### 2.5 Statistical analysis

Survival time was defined by the age of the subject in days. Events was defined as a subject’s first visit or admission to specialized services with the studied diagnosis. Observation of a subject’s data ended at the date they moved away from the country, the date of death, or the end of the follow-up on 31 December 2009. We used Cox proportional hazard regression models and we quantified the associations between the predictors and the outcomes with hazard ratios (HR) and their 95% confidence intervals. We calculated P-values using the Wald χ^2^ -test. In univariate analyses, we analyzed each predictor separately in a model. In multivariate analyses, we entered all defined predictors at once into the model.

We conducted a statistical analysis with SAS for Windows, version 9.4 TS1M5 (SAS Institute Inc., Cary, NC, USA).

## 3 Results

**Table 1** shows the descriptive characteristics of the categorical explanatory variables at the age of 8, and prevalences of treated psychiatric disorder groups by age 29. According to Cox proportional hazard regression analysis, out of 2,717 males and 2,683 females, 12.4% and 12.8%, respectively, had at least one psychiatric disorder diagnosed in specialized services in adolescence or adulthood. The cumulative treatment prevalence for schizophrenia and other non-affective psychoses was 2.1% and 1.2% for males and females, respectively; for bipolar disorder it was 0.5% and 0.9%, for depression it was 4.7% and 6.3%, for anxiety disorders it was 5.0% and 5.6%, for substance use disorders it was 3.7% and 1.7%, and for eating disorders among females it was 1.8%. Due to the low number of male participants with an eating disorder (n<5), associations between childhood factors were only studied for females with an eating disorder.

**Table 1:** Distribution of predictors at age 8 and psychiatric disorders diagnosed in specialized care between age 16 and 29^a^.

Predictor	Total		Any psychiatric disorder		Schizophrenia and non-affective psychoses		Bipolar disorder		Depression		Anxiety disorders		Substance-related disorders		Eating disorders^b^
	Males	Females	Males	Females	Males	Females	Males	Females	Males	Females	Males	Females	Males	Females	Females
	No.	No.	No. (%)	No. (%)	No. (%)	No. (%)	No. (%)	No. (%)	No. (%)	No. (%)	No. (%)	No. (%)	No. (%)	No. (%)	No. (%)
Total sample	2,717	2,683	336 (12.4)	347 (12.8)	57 (2.1)	33 (1.2)	13 (0.5)	25 (0.9)	127 (4.7)	172 (6.3)	135 (5.0)	153 (5.6)	101 (3.7)	47 (1.7)	48 (1.8)
Parental education level															
Upper secondary	918	901	85 (9.3)	107 (11.9)	26 (2.8)	14 (1.6)	3 (0.3)	8 (0.9)	37 (4.0)	53 (5.9)	33 (3.6)	41 (4.6)	19 (2.1)	10 (1.1)	18 (2.0)
Lower	1,658	1,634	228 (13.8)	219 (13.4)	26 (1.6)	18 (1.1)	9 (0.5)	17 (1.0)	84 (5.1)	111 (6.8)	95 (5.7)	105 (6.4)	71 (4.3)	32 (2.0)	26 (1.6)
Family structure															
Two biological parents	2,180	2,155	232 (10.6)	250 (11.6)	37 (1.7)	23 (1.1)	10 (0.5)	15 (0.7)	95 (4.4)	128 (5.9)	97 (4.4)	109 (5.1)	61 (2.8)	21 (1.0)	38 (1.8)
Other	433	410	89 (20.6)	80 (19.5)	16 (3.7)	9 (2.2)	2 (0.5)	10 (2.4)	29 (6.7)	37 (9.0)	32 (7.4)	37 (9.0)	35 (8.1)	21 (5.1)	8 (2.0)
School performance															
Average or better	2,238	2,338	264 (11.8)	295 (12.6)	48 (2.1)	30 (1.3)	11 (0.5)	23 (1.0)	99 (4.4)	150 (6.4)	111 (5.0)	134 (5.7)	76 (3.4)	39 (1.7)	44 (1.9)
Poor	423	250	68 (16.1)	34 (13.6)	9 (2.1)	1 (0.4)	2 (0.5)	1 (0.4)	27 (6.4)	12 (4.8)	23 (5.4)	12 (4.8)	24 (5.7)	3 (1.2)	0 (0.0)
Conduct problems															
<50th percentile	1,338	1,425	**104 (7.8)**	**158 (11.1)**	16 (1.2)	13 (0.9)	2 (0.1)	11 (0.8)	**45 (3.4)**	**84 (5.9)**	47 (3.5)	67 (4.7)	**20 (1.5)**	**19 (1.3)**	20 (1.4)
50th-90th percentile	999	825	**144 (14.4)**	**113 (13.7)**	29 (2.9)	10 (1.2)	7 (0.7)	8 (1.0)	**50 (5.0)**	**54 (6.5)**	59 (5.9)	55 (6.7)	**48 (4.8)**	**10 (1.2)**	20 (2.4)
>90th percentile	265	298	**76 (28.7)**	**55 (18.5)**	12 (4.5)	8 (2.7)	3 (1.1)	5 (1.7)	**29 (10.9)**	**24 (8.1)**	25 (9.4)	23 (7.7)	**29 (10.9)**	**12 (4.0)**	5 (1.7)
ADHD problems															
<50th percentile	1,297	1,739	**116 (8.9)**	**202 (11.6)**	25 (1.9)	18 (1.0)	5 (0.4)	19 (1.1)	**52 (4.0)**	**108 (6.2)**	44 (3.4)	87 (5.0)	28 (2.2)	22 (1.3)	32 (1.8)
50th-90th percentile	1,025	518	**138 (13.5)**	**76 (14.7)**	21 (2.0)	8 (1.5)	5 (0.5)	1 (0.2)	**43 (4.2)**	**31 (6.0)**	58 (5.7)	37 (7.1)	43 (4.2)	11 (2.1)	7 (1.4)
>90th percentile	260	267	**65 (25.0)**	**43 (16.1)**	11 (4.2)	5 (1.9)	2 (0.8)	3 (1.1)	**28 (10.8)**	**19 (7.1)**	26 (10.0)	20 (7.5)	24 (9.2)	8 (3.0)	4 (1.5)
Emotional symptoms															
<50th percentile	1,487	1,483	138 (9.3)	152 (10.2)	21 (1.4)	14 (0.9)	7 (0.5)	8 (0.5)	51 (3.4)	69 (4.7)	59 (4.0)	65 (4.4)	46 (3.1)	17 (1.1)	24 (1.6)
50th-90th percentile	817	788	123 (15.1)	121 (15.4)	22 (2.7)	12 (1.5)	4 (0.5)	14 (1.8)	49 (6.0)	68 (8.6)	42 (5.1)	54 (6.9)	31 (3.8)	16 (2.0)	16 (2.0)
>90th percentile	288	256	59 (20.5)	49 (19.1)	13 (4.5)	5 (2.0)	1 (0.3)	2 (0.8)	23 (8.0)	24 (9.4)	28 (9.7)	24 (9.4)	18 (6.3)	7 (2.7)	5 (2.0)
Self-reported depressive symptoms															
<50th percentile	1,449	1,370	135 (9.3)	147 (10.7)	27 (1.9)	16 (1.2)	6 (0.4)	11 (0.8)	48 (3.3)	77 (5.6)	56 (3.9)	67 (4.9)	40 (2.8)	22 (1.6)	28 (2.0)
50th-90th percentile	885	922	124 (14.0)	126 (13.7)	21 (2.4)	9 (1.0)	6 (0.7)	5 (0.5)	46 (5.2)	63 (6.8)	60 (6.8)	50 (5.4)	32 (3.6)	13 (1.4)	10 (1.1)
>90th percentile	312	280	70 (22.4)	55 (19.6)	9 (2.9)	6 (2.1)	1 (0.3)	8 (2.9)	32 (10.3)	23 (8.2)	19 (6.1)	29 (10.4)	26 (8.3)	7 (2.5)	6 (2.1)

a Bold face indicates a statistically significant predictor × sex interactions: conduct × sex predicting any psychiatric disorder (p <.001); hyperkinetic × sex predicting any psychiatric disorder (p <.001); conduct × sex predicting depression (p = .03); ADHD × sex predicting depression (p = .03); and conduct × sex predicting substance-related disorders (p = .02). Further analyses for these outcomes are sex-stratified.

b Frequencies and percentages are shown for females only, as only one male had been diagnosed with an eating disorder.

There were statistically significant interactions between conduct problems x sex (p<.001) and ADHD problems x sex (p<.001) with any psychiatric disorder, conduct problems x sex (p=.03) and ADHD problems x sex (p=.03) with depression, and finally conduct problems x sex with substance abuse disorders (p=.02). Further analysis of these outcomes was sex stratified.

**Table 2** shows the unadjusted associations between family demographics, school performance, and child psychopathology at age 8 and having any or a specific psychiatric diagnosis in specialized services. Among both genders, all psychopathology domains at age 8 (conduct, ADHD, anxiety, self-reported depression) and living in a non-intact family were associated with having any psychiatric disorder in adulthood. A low parental education level and poor school performance were associated with any psychiatric disorder only among males. Overall, childhood psychopathology was associated with the most specific psychiatric diagnoses. However, distinctive from other psychiatric diagnostic groups, eating disorders were not associated with any childhood risk factors, including parental education level, family structure, school performance, and childhood psychopathology. **Supplementary Table 1** shows that all the psychopathology domains were associated with specialized psychiatric service use when they were analyzed as linear scales. Similarly, to the categorical analysis, the presence of an eating disorder was the only outcome that was not predicted by any psychiatric problem at age 8.

**Table 2:** Single predictor associations between variables at age 8 and psychiatric disorders diagnosed in specialized care between age 16 and 29^a^.

	Any psychiatric diagnosis		Schizophrenia and non-affective psychoses	Bipolar disorder	Depression		Anxiety disorders	Substance-related disorders		Eating disorders^d^
	Males^b^	Females^b^	Both sexes	Both sexes	Males^b^	Females^b^	Both sexes	Males^b^	Females^b^	Females
Predictor	HR (95% CI)	HR (95% CI)	HR (95% CI)^c^	HR (95% CI)^c^	HR (95% CI)	HR (95% CI)	HR (95% CI)^c^	HR (95% CI)	HR (95% CI)	HR (95% CI)^c^
Sex										
Male	–	–	**1.7 (1.1 - 2.6)**	0.5 (0.3 - 0.995)	–	–	0.9 (0.7 - 1.1)	–	–	–
Parental education level										
Lower than upper secondary	**1.5 (1.2 - 2.0)**	1.1 (0.9 - 1.4)	0.6 (0.4 - 0.9)	1.3 (0.6 - 2.6)	1.3 (0.9 - 1.9)	1.2 (0.8 - 1.6)	**1.5 (1.2 - 2.0)**	**2.1 (1.3 - 3.5)**	1.8 (0.9 - 3.6)	0.8 (0.4 - 1.4)
Family structure										
Other than two biological parents	**2.1 (1.6 - 2.6)**	**1.8 (1.4 - 2.3)**	**2.2 (1.4 - 3.4)**	**2.5 (1.3 - 5.0)**	**1.6 (1.03 - 2.4)**	**1.6 (1.1 - 2.2)**	**1.8 (1.3 - 2.3)**	**3.0 (2.0 - 4.5)**	**5.4 (2.9 - 9.8)**	1.1 (0.5 - 2.4)
School performance										
Poorer than average	**1.4 (1.1 - 1.8)**	1.1 (0.8 - 1.5)	0.8 (0.4 - 1.6)	0.7 (0.2 - 2.2)	1.5 (0.96 - 2.2)	0.7 (0.4 - 1.3)	1.0 (0.7 - 1.4)	**1.7 (1.1 - 2.7)**	0.7 (0.2 - 2.3)	N/A
Conduct problems										
50th-90th percentile	**1.9 (1.5 - 2.5)**	1.3 (0.98 - 1.6)	**2.0 (1.2 - 3.2)**	1.8 (0.9 - 3.9)	**1.5 (1.01 - 2.3)**	1.1 (0.8 - 1.6)	**1.5 (1.2 - 2.0)**	**3.3 (2.0 - 5.6)**	0.9 (0.4 - 1.9)	1.7 (0.9 - 3.2)
>90th percentile	**4.1 (3.1 - 5.5)**	**1.7 (1.3 - 2.4)**	**3.5 (2.0 - 6.2)**	**3.0 (1.2 - 7.2)**	**3.4 (2.1 - 5.4)**	1.4 (0.9 - 2.2)	**2.1 (1.5 - 3.0)**	**7.6 (4.3 - 13.5)**	**3.0 (1.5 - 6.3)**	1.2 (0.4 - 3.2)
ADHD problems										
50th-90th percentile	**1.6 (1.2 - 2.0)**	1.3 (0.99 - 1.7)	1.2 (0.7 - 1.9)	0.6 (0.2 - 1.4)	1.1 (0.7 - 1.6)	1.0 (0.6 - 1.4)	**1.5 (1.2 - 2.0)**	**2.0 (1.2 - 3.2)**	1.7 (0.8 - 3.5)	0.7 (0.3 - 1.7)
>90th percentile	**3.1 (2.3 - 4.2)**	**1.4 (1.02 - 2.0)**	**2.1 (1.2 - 3.7)**	1.3 (0.5 - 3.3)	**2.8 (1.8 - 4.4)**	1.2 (0.7 - 1.9)	**2.1 (1.5 - 3.0)**	**4.5 (2.6 - 7.7)**	**2.4 (1.1 - 5.3)**	0.8 (0.3 - 2.3)
Emotional symptoms										
50th-90th percentile	**1.7 (1.3 - 2.1)**	**1.5 (1.2 - 2.0)**	**1.8 (1.1 - 2.9)**	**2.2 (1.1 - 4.4)**	**1.8 (1.2 - 2.6)**	**1.9 (1.3 - 2.6)**	**1.4 (1.1 - 1.9)**	1.2 (0.8 - 1.9)	1.8 (0.9 - 3.5)	1.3 (0.7 - 2.4)
>90th percentile	**2.4 (1.8 - 3.3)**	**2.0 (1.4 - 2.7)**	**2.8 (1.6 - 5.0)**	1.1 (0.3 - 3.9)	**2.4 (1.5 - 3.9)**	**2.1 (1.3 - 3.3)**	**2.4 (1.7 - 3.3)**	**2.0 (1.2 - 3.5)**	2.4 (0.997 - 5.8)	1.2 (0.5 - 3.2)
Self-reported depressive symptoms										
50th-90th percentile	**1.5 (1.2 - 2.0)**	**1.3 (1.01 - 1.6)**	1.1 (0.7 - 1.8)	1.0 (0.5 - 2.1)	**1.6 (1.1 - 2.4)**	1.2 (0.9 - 1.7)	**1.4 (1.1 - 1.8)**	1.3 (0.8 - 2.1)	0.9 (0.4 - 1.7)	0.5 (0.3 - 1.1)
>90th percentile	**2.6 (1.9 - 3.4)**	**1.9 (1.4 - 2.7)**	1.7 (0.9 - 3.0)	**2.6 (1.2 - 5.8)**	**3.2 (2.1 - 5.0)**	1.5 (0.9 - 2.4)	**1.9 (1.4 - 2.6)**	**3.1 (1.9 - 5.1)**	1.6 (0.7 - 3.7)	1.1 (0.4 - 2.5)

HR, hazard ratio; CI, confidence interval; N/A, not applicable.

a Bold face indicates association significant at p <.05 in the Cox regression model.

b Results are sex-stratified as the following significant sex × predictor interactions were present (see footnote a in **Table 1** for details).

c Adjusted for sex.

d Results are shown for females only, as only one male had been diagnosed with an eating disorder.

**Table 3** shows the results of adjusted analyses including all variables associated with specific outcomes at a level of 0.1 significance. Living in a non-intact family independently predicted any psychiatric disorder (males, HR=1.5, 95% CI =1.1-1.9; females, HR=1.6, 95% CI = 1.2-2.1), psychotic disorder, bipolar disorder, anxiety disorders and substance abuse disorder. However, this factor was only associated with female depression outcomes, not male. Interestingly, family structure was the only childhood factor predicting female substance use disorder. School performance did not predict any outcome in multivariate analyses. The only association with parental education level was found between higher parental education level and psychosis group. Conduct, anxiety, and depression measures at age 8 predicted any psychiatric outcomes, while ADHD problems did not predict any of the outcomes when controlled with other psychiatric problems and family variables.

**Table 3:** Multivariate models of variables at age 8 and psychiatric disorders diagnosed in specialized care between age 16 and 28^a^.

	Any psychiatric diagnosis		Schizophrenia and non-affective psychoses	Bipolar disorder	Depression		Anxiety disorders	Substance-related disorders	
	Males	Females	Both sexes	Both sexes	Males	Females	Both sexes	Males	Females
Predictor	HR (95% CI)	HR (95% CI)	HR (95% CI)	HR (95% CI)	HR (95% CI)	HR (95% CI)	HR (95% CI)	HR (95% CI)	HR (95% CI)
Sex									
Male	–	–	1.6 (0.99 - 2.5)	0.5 (0.2 - 0.9)	–	–	0.8 (0.6 - 1.1)	–	–
Parental education level									
Lower than upper secondary	1.2 (0.9 - 1.6)	–	0.5 (0.3 - 0.7)	–	–	–	1.3 (0.98 - 1.7)	1.5 (0.9 - 2.6)	–
Family structure									
Other than two biological parents	**1.5 (1.1 - 1.9)**	**1.6 (1.2 - 2.1)**	**1.8 (1.1 - 3.0)**	**2.2 (1.1 - 4.4)**	1.1 (0.7 - 1.8)	**1.5 (1.1 - 2.2)**	**1.4 (1.1 - 1.9)**	**2.0 (1.2 - 3.2)**	**4.2 (2.2 - 8.0)**
School performance									
Poorer than average	1.0 (0.7 - 1.3)	–	–	–	1.1 (0.7 - 1.8)	–	–	1.1 (0.7 - 1.9)	–
Conduct problems									
50th–90th percentile	**1.6 (1.2 - 2.1)**	1.2 (0.9 - 1.5)	**2.0 (1.2 - 3.4)**	1.6 (0.8 - 3.5)	1.4 (0.9 - 2.1)	–	1.3 (0.99 - 1.7)	**2.7 (1.5 - 4.9)**	0.7 (0.3 - 1.5)
>90th percentile	**2.5 (1.6 - 3.7)**	**1.5 (1.01 - 2.1)**	**3.3 (1.6 - 7.0)**	2.2 (0.9 - 5.5)	1.8 (0.95 - 3.6)	–	1.3 (0.8 - 2.0)	**3.9 (1.8 - 8.4)**	1.8 (0.7 - 4.4)
ADHD problems									
50th–90th percentile	1.0 (0.8 - 1.4)	1.0 (0.7 - 1.3)	0.8 (0.5 - 1.4)	–	0.7 (0.5 - 1.1)	–	1.2 (0.9 - 1.6)	1.0 (0.6 - 1.8)	1.2 (0.6 - 2.7)
>90th percentile	1.2 (0.8 - 1.8)	1.0 (0.6 - 1.4)	0.9 (0.4 - 1.9)	–	1.2 (0.6 - 2.3)	–	1.3 (0.8 - 1.9)	1.2 (0.5 - 2.4)	1.2 (0.5 - 3.3)
Emotional symptoms									
50th–90th percentile	**1.4 (1.1 - 1.8)**	**1.4 (1.1 - 1.8)**	1.5 (0.9 - 2.5)	2.0 (0.98 - 3.9)	1.5 (0.97 - 2.2)	**1.9 (1.3 - 2.6)**	**1.3 (1.02 - 1.8)**	0.9 (0.6 - 1.5)	1.6 (0.8 - 3.1)
>90th percentile	**1.5 (1.1 - 2.1)**	**1.7 (1.2 - 2.4)**	**2.0 (1.1 - 3.7)**	0.8 (0.2 - 2.9)	**1.7 (1.01 - 2.8)**	**2.0 (1.3 - 3.2)**	**2.0 (1.4 - 2.8)**	1.2 (0.6 - 2.1)	1.9 (0.8 - 4.6)
Self-reported depressive symptoms									
50th–90th percentile	1.2 (0.9 - 1.6)	1.2 (0.9 - 1.5)	–	0.8 (0.4 - 1.8)	1.4 (0.9 - 2.1)	–	1.2 (0.9 - 1.5)	0.9 (0.6 - 1.5)	–
>90th percentile	**1.7 (1.2 - 2.3)**	**1.6 (1.1 - 2.2)**	–	2.0 (0.9 - 4.7)	**2.5 (1.5 - 4.1)**	–	1.4 (0.98 - 2.0)	1.7 (0.97 - 3.0)	–

HR, hazard ratio; CI, confidence interval.

a Variables with p-values < 0.1 in univariate models were included in the multivariate Cox regression models. Bold face indicates associations with p < 0.05 in the multivariate models.

In multivariate analyses, conduct problems at age 8 predicted psychotic disorders and male substance use disorder. Anxiety at age 8 predicted psychotic disorders, anxiety, and depression. Self-reported depressive symptoms predicted male depression, while association with anxiety disorders was close to significant. In most cases, it was the group scoring above the 90th percentile in childhood symptom levels that predicted specific psychiatric outcomes. However, in the conduct scale, even the lower symptom level—those who scored between the 50th and 90th percentile—predicted psychotic disorders and male substance use disorders, compared with the reference group scoring below the 50th percentile. Similarly, in the anxiety and depression scales, lower levels of scores at age 8 were associated with later anxiety and female depression diagnoses.

**Figure 2:**
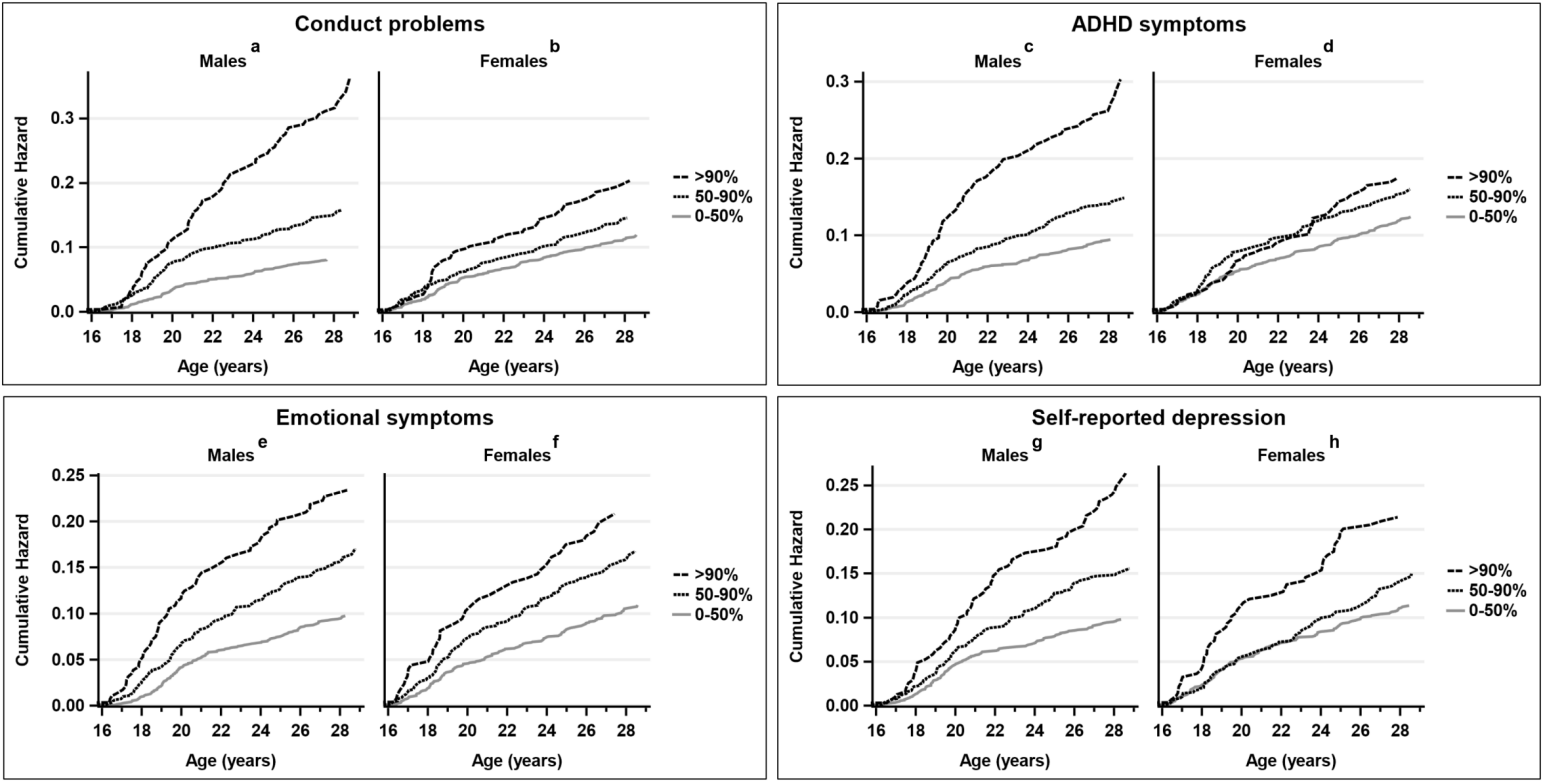
Cumulative hazard of any psychiatric diagnosis in specialized care between age 16 and 29 according to the <50th percentile, 50th-90th percentile, and >90th percentile of sex-specific conduct problems, ADHD symptoms, emotional symptoms, and self-reported depression at age 8 among males **(a, c, e, g)** and females **(b, d, f, h)**.

**Figure 2** depicts the cumulative hazard of any psychiatric diagnosis in specialized care between age 16 and 29 according to the <50th percentile, 50th–90th percentile and >90th percentile of sex-specific conduct problems, ADHD problems, anxiety, and self-reported depressive symptoms at age 8. Because of the strong association between conduct problems and later service use, this relationship was further analyzed. Among males (**Figure 2a**) and females (**Figure 2b**) with high levels of conduct problems, the product-limit survival estimate (first service use because of any psychiatric disorder) depreciated with longer follow-up times. The difference between those scoring above the 90th percentile and between the 50th and 90th percentiles, when compared with males scoring below 50th percentile, was significant (HR 4.1, 95%CI 3.1-5.5; HR 1.9, 95%CI 1.5-2-5). Among females, the findings were more modest and only significant for those above the 90th percentile (HR, 1.7, 95%CI 1.3-2.4; HR 1.3, 95%CI 0.98-1.6, respectively).

## 4 Discussion

We examined the association between multi-informant ratings of psychiatric symptoms in middle childhood and specialized health care service use in adolescence and young adulthood in a large Finnish population-based sample. The first main finding was the strong association between parent reports of conduct problems, especially those for males, with adult psychiatric service use and particularly with psychotic and substance use disorders. Second, childhood anxiety and depressive problems were associated with psychiatric service use for similar problems in adulthood, referring to homotypic continuity. Somewhat unexpectedly, we found no independent association between childhood ADHD problems and adult psychiatric service use in the final model. Finally, living in a non-intact family was an unspecific factor associated with most psychiatric diagnostic groups.

Children with conduct problems are at an increased risk of developing life-long disorders related to mood, anxiety, impulse-control, psychoses, smoking, substance abuse, and poor resilience (2, 29, 31, 32). In the present study, a novel discovery was that childhood conduct problems were specifically associated with service use related to psychotic and substance use disorders, which are among the most costly, chronic, and life-threatening health problems. The polygenic risk for schizophrenia associates with oppositional defiant disorder/conduct disorder (ODD/CD) (33), and there is evidence for both common and specific risk factors for schizophrenia and violence, ranging from genetic variants linked to stress regulation and adversity (34) to neurodevelopmental deficits (35). Birth or genetic high‐risk cohort studies have shown that deficits in total IQ and motor performance precede the prodrome and the onset of psychotic illness (36), and among high‐risk individuals, cognitive deficits in verbal fluency and memory functioning are associated with transition to psychosis (37). Deficits in the verbal domain are common among adolescents with severe conduct problems (38).

Previous studies have shown an association between early-onset persistent (EOP) conduct problems and various forms of later problems (11). In a meta-analysis conducted by Bevilaqcua et al. (39), the finding was repeated: seven separate studies supported the finding that young adults in the early-onset persistent group had the highest risk for mental health problems, including depression, compared to adolescent-onset and childhood-limited groups. Our findings are in line with previous cohort study findings (11, 27, 40). It must be noted that, in our study, symptoms were only evaluated when the children were 8 years old and the data was registered as a response; thus, symptoms related to onset in adolescence were not included in this study. This affects the comparison of our results with the findings from the previous studies, in which symptoms have also been evaluated in adolescence. However, we analyzed the 50th–90th percentile symptom category as a separate variable, and even this lower symptom category predicted male psychotic disorders and male substance-use disorders.

The strong association between early childhood conduct problems and later psychiatric service use highlights the importance of identifying early-onset conduct problems and providing early interventions for targeted high-risk groups. There is mounting evidence from randomized controlled trials (RCT) that parent training for childhood disruptive behavior problems reduces children’s behavioral problems and improves parenting skills (41). Our findings highlight the significance and potential cost-effectiveness of parent training, showing that it has the potential to decrease psychopathology in adulthood.

Among the internalizing disorders, a trend of homotypic continuity was found. Adult-assessed anxiety at age 8 were independently associated with anxiety and depression in adulthood. Self-reported depressive symptoms were independently associated with male depression, and the association with anxiety disorders was close to significant. These findings support previous studies showing that childhood depression and anxiety predict their adult counterparts (4, 5 8), and cross-predict each other (3–5, 7, 8). As it is known that mood disorders beginning in childhood are associated with various risk factors and higher comorbidity, compared to adult-onset disorders, as well as poorer prognosis in adulthood (42, 43), this further emphasizes the importance of effectively recognizing and treating these problems among children.

Previous findings on the relationship between childhood ADHD problems and adolescent and adult psychiatric outcomes are somewhat inconsistent. For example, while some follow-up studies show that children with ADHD are at an increased risk of developing antisocial disorders (44, 45), some studies suggest that ADHD, in the absence of conduct problems, is not a developmental precursor of later antisociality (46, 47). In our previous report, ADHD symptoms at age 8 predicted psychiatric hospital treatment and several psychiatric diagnoses among males by age 24 in the univariate analyses, and no statistically significant associations were found in the multivariate analyses (48). This further supports findings from some previous longitudinal population-based studies where attention problems have been adjusted for other childhood problems, especially with comorbid conduct problems (49–51). Of note, the use of psychostimulants was almost nonexistent in Finland until the end of the 1990s (52). Therefore, the results have not been influenced by possible positive effects of the early use of psychostimulants on long-term outcomes among children with attention problems.

While all other psychiatric disorder service groups were found to be associated with family or child psychopathology factors, no associations were found between the group of females with eating disorders and childhood factors. However, the rather small number of eating disorder cases should be considered when interpreting these results.

Living in a non-nuclear family at the age of 8 predicted all studied psychiatric diagnoses, as only depression among males was not associated with family structure in childhood. Previous studies have shown that single parenthood (53) and parental divorce or separation (54) are risk factors for children’s later psychopathology (53–55), up until early adulthood (53, 55). Living in a non-nuclear family may be associated with economic hardship, low social support, conflicts between the parents, maltreatment, as well as problems in parental practices, adjustment, and mental health (56, 57). When interpretating the results of this study, it is important to note the remarkable changes in society during recent decades, including a higher education level among women, the practice of joint physical custody becoming more normative, and children living in a non-nuclear family becoming less stigmatized. Unfortunately, in this study it was only possible to study the impact of the family structure on later psychiatric diagnoses, and factors that might be associated with the non-intact family structure, such as reasons leading to separation of the parents, or socioeconomic hardship, could not be taken into account. More research on associations between family structure, which has become even more complex in modern society, and child mental health is warranted.

One of the major challenges in child and adolescent psychiatric assessment is that multiple informants are needed to gather information about the child’s symptoms. A special feature of this study is combining information from three different sources (child, parent and teacher) as early as at the age of 8. The Finnish 1981 birth cohort data has been utilized in several previous publications, and differences among informants in this sample have been reported by Almqvist et al. (58). Their findings were in line with previous reports, which have shown low agreement between parent, teacher and child ratings, while they all show predictive association with later adversities (50, 59–61).

It is worth noting that several diagnostic groups in this study showed not only homotypic, but also heterotypic continuity. Considering the young age of the participants in this study, these findings highlight the importance of offering evidence-based interventions for both internalizing and externalizing disorders at an early age. Clinicians and policy makers should be aware that children with mental health concerns are at risk of developing many different forms of psychiatric comorbidity in adulthood, and that the range of symptoms can change its form in the course of time. Since our study suggested that a non-intact family structure may increase the risk of later mental health challenges, it is important to pay particular attention to children who have experienced parental loss or separation at an early age. Any efforts aimed at supporting these children and their families may help ease the psychological burden they could face later in life.

### 4.1 Limitations

The study has several limitations to be considered when interpretating the results. First, caseness at baseline was based solely on symptom scales. However, including combined information from parents and teachers increases the validity of detected problems among children. Given the large number of subjects, the rating scale approach at baseline provides useful information but lacks the specificity and additional depth that more formally structured interviews would provide. Second, information on psychiatric diagnoses in adulthood was based on registers and not monitored assessment procedures (e.g., structured diagnostic interviews). However, Finnish specialized health care registers have been shown to have high validity in regarding disorders such as schizophrenia, bipolar disorder, autism, and ADHD (62, 63). It must also be noted that register-based information about outpatient service use was not available in Finland before year 1998. Therefore, the follow-up started from age 16. It is most likely that the majority of children and adolescents who had been referred to specialized psychiatric services between age 8 and 15 also had contacts to specialized psychiatric services later in life. Because register data was not available during the years when children born 1981 were 8–15 years old we are not able to give register-based information about contacts to specialized services during that time period. Although the unmet needs and low service use are still significant, the use of services was significantly lower 20 to 30 years ago, as shown in our time-trend studies (64, 65).

Third, it is important to note that in the any psychiatric diagnosis group, patients with intellectual disability (F70-F79) were also included. Patients with these diagnoses often have comorbid diagnoses, which may have affected the study results. Fourth, we were not able to examine the regional differences in the availability of services and their impact on the results. It is possible that some patients who would have needed services have not reached them because of regional lack of services. It has also been previously reported that hospitalization because of psychiatric disorders is more likely among patients from lower SES (66). Thus, the incidence of service use must not be interpreted to be the same as the incidence of the examined disorders. Additionally, when we examined the impact of the family structure, it must be noted that in depth information about childhood family and other environmental factors (e.g, parental psychopathologic disorder, parenting style, and family environment) was lacking. We must also consider attrition as a potential source of bias; however, the attrition rate at baseline was only 3.4% of the sample in 1989, and the number of parents who refused to participate in the study was exceptionally low. No information is available of these children and their parents who either refused to participate or could not be contacted. The missing data was due to incorrect documentation of the PIN codes and not related to child or parent characteristics.

## 5 Conclusion

Children with conduct problems are at an increased risk of developing life-long serious disorders, such as psychotic disorders and substance abuse problems, and these children should be effectively screened and treated for psychiatric disorders. Continuity among anxiety and mood disorders is considerable when child self-reports are also included, and adequate treatment of these problems could reduce morbidity in adulthood. The findings emphasize a need of a multi-informant approach in early identification and treatment planning. Importantly, the study also shows that even brief scales used only one time during a child’s development can have strong predictive value for later adversities. This indicates that many children at risk could be rather easily identified in health check-ups or school settings. This is important because sufficient evidence shows that parent training for disrupting behavioral problems and cognitive behavioral therapy for internalizing problems are effective. Further implementation of these treatments in society has the potential to relieve both human suffering and enormous economic burden.

## Data Availability

The datasets presented in this article are not readily available because of GDPR regulations and confidentiality. Requests to access the datasets should be directed to Andre Sourander, andsou@utu.fi.
