# Supplementary material for: Psychiatric symptoms at age 8 as predictors of specialized health service use for psychiatric disorders in late adolescence and early adulthood: findings from the Finnish Nationwide 1981 Birth Cohort Study
Source: Front Psychiatry. 2025 Jul 1;16:1600022. doi: 10.3389/fpsyt.2025.1600022 (PMC12261452; doi:10.3389/fpsyt.2025.1600022)
Supplement: Supplementary file 1 [file Table1.docx]

Supplementary Material

# Supplementary Figures and Tables

**Supplementary Table 1**. Associations between symptom categories at age 8 and psychiatric disorders diagnosed in specialized care between age 16 and 29, linear analysis.^a^

|  | Any psychiatric diagnosis | | Schizophrenia and non-affective psychoses | Bipolar disorder | Depression | | Anxiety disorders | Substance-related disorders | | Eating disorders^d^ |
| --- | --- | --- | --- | --- | --- | --- | --- | --- | --- | --- |
|  | Males^b^ | Females^b^ | Both sexes | Both sexes | Males^b^ | Females^b^ | Both sexes | Males^b^ | Females^b^ | Females |
| Predictor | HR (95% CI) | HR (95% CI) | HR (95% CI)^c^ | HR (95% CI) | HR (95% CI) | HR (95% CI) | HR (95% CI)^c^ | HR (95% CI) | HR (95% CI) | HR (95% CI)^c^ |
| Conduct problems | **1.5 (1.4-1.7)** | **1.2 (1.08-1.3)** | **1.4 (1.2-1.6)** | **1.3 (1.04-1.6)** | **1.4 (1.3-1.7)** | 1.1 (0.96-1.3) | **1.3 (1.2-1.5)** | **1.8 (1.6-2.1)** | **1.4 (1.1-1.6)** | 1.1 (0.8-1.4) |
| ADHD problems | **1.4 (1.3-1.6)** | **1.1 (1.02-1.2)** | 1.1 (0.97-1.4) | 1.03 (0.7-1.4) | **1.4 (1.2-1.6)** | 1.05 (0.9-1.2) | **1.2 (1.1-1.4)** | **1.6 (1.4-1.9)** | 1.2 (0.98-1.5) | 0.9 (0.7-1.3) |
| Emotional symptoms | **1.4 (1.3-1.5)** | **1.3 (1.2-1.4)** | **1.4 (1.2-1.7)** | 1.3 (0.95-1.7) | **1.4 (1.2-1.6)** | **1.3 (1.1-1.5)** | **1.3 (1.2-1.5)** | **1.2 (1.1-1.5)** | **1.4 (1.1-1.7)** | 1.00 (0.8-1.3) |
| Self-reported depressive symptoms | **1.4 (1.3-1.5)** | **1.3 (1.2-1.4)** | **1.2 (1.03-1.5)** | **1.4 (1.1-1.8)** | **1.4 (1.3-1.6)** | **1.3 (1.1-1.5)** | **1.3 (1.1-1.4)** | **1.4 (1.2-1.7)** | 1.2 (0.9-1.5) | 1.01 (0.7-1.4) |

Abbreviations: HR, hazard ratio; CI, confidence interval; N/A, not applicable.

^a^ Bold face indicates association significant at p < .05 in the Cox regression model.

^b^ Results are sex-stratified as the following significant sex × predictor interactions were present (see footnote a in Table 1 for details).

^c^ Adjusted for sex.

^d^ Results are shown for females only, as only one male had been diagnosed with an eating disorder.
